# Heterogeneity of Circulating Influenza Viruses and Their Impact on Influenza Virus Vaccine Effectiveness During the Influenza Seasons 2016/17 to 2018/19 in Austria

**DOI:** 10.3389/fimmu.2020.00434

**Published:** 2020-03-17

**Authors:** Monika Redlberger-Fritz, Michael Kundi, Therese Popow-Kraupp

**Affiliations:** ^1^Centre of Virology, Medical University Vienna, Vienna, Austria; ^2^Department of Environmental Health, Medical University Vienna, Vienna, Austria

**Keywords:** influenza vaccine effectiveness, influenza genetic heterogeneity, influenza virus, genetic variability and vaccine effectiveness, influenza antigenic drift and influenza vaccine effectiveness

## Abstract

The constantly changing pattern in the dominance of viral strains and their evolving subclades during the seasons substantially influences influenza vaccine effectiveness (IVE). In order to further substantiate the importance of detailed data of genetic virus characterization for IVE estimates during the seasons, we performed influenza virus type and subtype specific IVE estimates. IVE estimates were assessed using a test-negative case-control design, in the context of the intraseasonal changes of the heterogeneous mix of circulating influenza virus strains for three influenza seasons (2016/17 to 2018/19) in Austria. Adjusted overall IVE over the three seasons 2016/17, 2017/18, and 2018/19 were −26, 39, and 63%, respectively. In accordance with the changing pattern of the circulating strains a broad range of overall and subtype specific IVEs was obtained: A(H3N2) specific IVE ranged between −26% for season 2016/17 to 58% in season 2018/19, A(H1N1)pdm09 specific IVE was 25% for the season 2017/18 and 65% for the season 2018/19 and Influenza B specific IVE for season 2017/18 was 45%. The results obtained in our study over the three seasons demonstrate the increasingly complex dynamic of the ever changing genetic pattern of the circulating influenza viruses and their influence on IVE estimates. This emphasizes the importance of detailed genetic virus surveillance for reliable IVE estimates.

## Introduction

Each year, influenza epidemics infect about 5–10% of adults and 10–20% of children. Influenza causes febrile illnesses that range in severity from mild to severe and can lead to hospitalization and even death (1). The risk of developing these serious complications is aggravated especially in the very young and in the elderly. The most effective way to prevent influenza virus infection and associated complications is by vaccination (2, 3). Unfortunately, influenza viruses continually change over time through genetic and antigenic drift of their surface glycoproteins to escape virus neutralization by immune response. Therefore, the composition of the influenza vaccines has to be reconsidered annually and if required, revision is performed according to the most recent changes of the circulating strains (4). Despite the yearly update and revaccination, the ability of the vaccine to prevent influenza virus infection in the general population varies each year (2).

Immunity generated by influenza vaccines is a complex issue and is not only influenced by the match between vaccine strains and the circulating viruses, but is also affected by the vaccinee's individual immunological history like number and type of previous influenza virus infections and/or previous influenza virus vaccinations. In addition, waning of vaccine-induced immunity during the season is also described as a contributing factor causing suboptimal influenza vaccine effectiveness (IVE) (5), whereby the decrease of influenza virus vaccine specific antibodies as test variable was used only in few studies. The majority of studies describing type and subtype specific waning of vaccine induced immunity use decreasing intraseasonal IVE estimates, and consider time since vaccination as a test variable. The influence of changes in the dynamics of circulating influenza virus types/subtypes during the season and especially of their newly evolving drift variants are not sufficiently taken into consideration. The great influence of the constantly changing pattern of viral strains during the season and its impact on intraseasonal IVE estimates has been clearly demonstrated in previous studies (4, 6) and underscore the importance to perform IVE estimates in the context of detailed virus characterization. In order to further substantiate the importance to use detailed data of virus characterization during the season for IVE estimates, we performed influenza virus type and subtype specific IVE estimates overall and over time intervals during the seasons. Therefore, a test-negative case-control design was used, in the context of the intraseasonal changes of the heterogeneous mix of circulating influenza virus strains for three influenza seasons (2016/17 to 2018/19) in Austria.

## Materials and Methods

### Sentinel Influenza Surveillance System and Samples Tested

Sentinel surveillance for influenza viruses was performed as described previously (6). Briefly, annual influenza virus surveillance is performed from October (calendar week 40) through April (week 16 of the following year) and is based on sentinel physicians (general practitioners and pediatricians throughout Austria) forming part of the Diagnostic Influenza Network Austria (DINOE), who collect nasopharyngeal swabs from patients presenting with influenza like illness as defined by the ECDC (7). The samples are submitted to and analyzed by the NIC Austria, Centre of Virology, Medical University Vienna. Epidemiological information including information on age, gender, underlying health conditions (like diabetes, cardio-vascular diseases, chronic lung diseases, malignant diseases), adiposity, smoking habits, vaccination status, kind of vaccine used [trivalent inactivated vaccine (TIV), adjuvanted trivalent inactivated vaccine (aTIV), quadrivalent inactivated vaccine (QIV), or live attenuated vaccine (LAIV)], date of onset of symptoms and of specimen collection is provided with the sample (6).

### Vaccination

Available influenza vaccines for the season 2016/17 in Austria were: TIV and aTIV containing the following recommended vaccine strains: A(H3N2): A/Hong Kong/4801/2014-like virus, A(H1N1)pdm09: A/California/7/2009-like virus and influenza B: B/Brisbane/60/2008-like virus (Victoria lineage), the LAIV included additionally B/Phuket/3073/2013-like virus.

For the season 2017/18: TIV and aTIV vaccine strains were A(H3N2): A/Hong Kong/4801/2014-like virus, A(H1N1)pdm09: A/Michigan/45/2015-like virus and influenza B: B/Brisbane/60/2008-like virus (Victoria lineage). During the season 2017/18 quadrivalent inactivated influenza vaccines (QIV) were available in Austria for the first time. In addition to the 2017/18 TIV vaccine components QIV and LAIV included also the B/Phuket/3073/2012 (Yamagata lineage) strain.

During season 2018/19 TIV, aTIV, QIV, and LAIV were available. The used vaccine strains for this season were A(H3N2): A/Singapore/INFIMH-16-0019/2016-like virus, A(H1N1)pdm09: A/Michigan/45/2015-like virus and B/Colorado/06/2017-like virus (Victoria lineage), for the QIV and LAIV vaccines additionally B/ Phuket/3073/2013-like virus (Yamagata lineage) was included.

Influenza vaccination in Austria is usually carried out between calendar weeks 40 to 48 and the non-adjuvanted, inactivated influenza vaccines were primarily used. People above 65 years of age were preferentially vaccinated with MF095 adjuvanted trivalent inactivated vaccines. Children between 2 and 18 years were vaccinated either with LAIV or inactivated vaccines (TIV or QIV).

### Influenza Virus Detection and Genotyping

Influenza virus detection and genotyping of the HA- and NA-gene were performed as previously described (6). Briefly, sentinel specimens were tested for influenza viruses by reverse transcription realtime PCR (RT realtime PCR). After RNA extraction, amplification, and purification, sequencing was performed using an Applied Biosystems ABI 3130xl platform (8, 9). Phylogenetic and molecular evolutionary analyses were performed using software package MEGA Version 4 (10). “Kimura-2” distance method and “Neighbour-Joining” algorithm were used for the phylogenic tree reconstruction.

### Estimation of Influenza Vaccine Effectiveness

Overall IVE against laboratory confirmed influenza virus infections were estimated as described previously (6) by use of the test-negative case-control design where a case is defined as a patient with influenza as confirmed by RT-PCR and a control as a patient tested negative for influenza virus. Odds ratios (OR) for medically attended, laboratory-confirmed influenza were estimated by multivariate logistic regression adjusting for gender, age, and comorbidities as covariates. For age group specific estimates the age was excluded as potential confounder in the respective model. Calculations were done using the generalized linear model with binomial counts and logit link (SPSS 25.0, IBM Corporation, USA).

The IVE was calculated as (1-OR) x 100% to compare vaccination status of cases with controls.

Inclusion criteria for this analysis were: availability of complete information on the patient (age, gender, comorbidities, vaccination status), specimen collection within 7 days after onset of ILI symptoms, and at least 2 weeks between vaccination and onset of ILI symptoms. Patients not fulfilling the inclusion criteria and patients under the age of 6 month were excluded.

IVE estimates were calculated overall and type/subtype specific for the whole season and during different time periods of the season. A prerequisite for the IVE calculation was the epidemic influenza virus activity, as indicated by a rate of influenza positive samples of ≥50%. In addition overall and type/subtype specific IVE were also calculated for different age-groups (6 month to 14 years, 15–64 years and above 65 years).

A query on prior season vaccination status was included in the laboratory test form accompanying each sample, but an adjustment of the IVE estimation for the prior season vaccination status could not be performed, as the number of patients vaccinated in two or more consecutive seasons was too small for reliable estimates (<4% in our study population).

## Results

### Influenza Virus Activity in Austria During the Seasons 2016/17 and 2018/19

**Figure 1:**
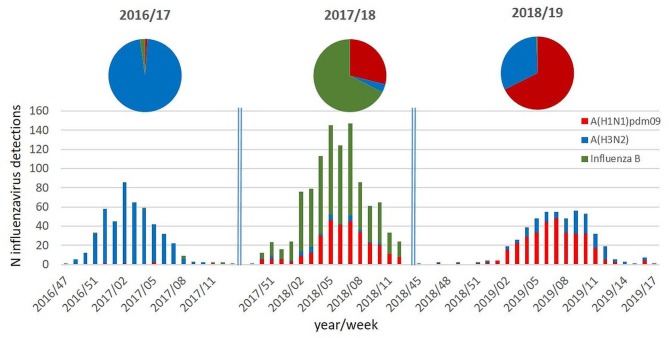
Columns: number of influenza virus detections per week and per type/subtype during the influenza seasons 2016/17 to 2018/19; pie chart: proportion (%) of the circulating influenza virus types/subtypes during the respective influenza seasons.

As can be seen in Figure 1, the three influenza seasons in Austria differed significantly with regard to the circulating types and subtypes and with regard to the intensity of influenza virus activity.

The season 2016/17 was a moderate influenza season with an influenza incidence of 270,000 cases in Austria and was characterized by the nearly exclusive circulation of influenza A(H3N2) viruses. The season 2017/18 was dominated by influenza B viruses and with co-circulation of A(H1N1)pdm09 viruses. Influenza A(H3N2) viruses were only detectable in a minority of samples. Despite the dominance of influenza B viruses, season 2017/18 was a very severe influenza season with an incidence of 440,000 influenza cases in Austria. In the following season 2018/19, again, a totally different pattern of virus circulation was observed. This season was dominated by A(H1N1)pdm09 viruses with co-circulating A(H3N2) viruses. The influenza incidence was 145,000 influenza cases in Austria, and it was therefore a moderate influenza season.

Detailed information on the epidemiologic characteristics of the three influenza seasons (the number of samples tested, number of influenza viruses detected and detailed virus characterization results) are summarized in Table 1.

**Table 1:** Epidemiologic characteristics of the three influenza seasons: number of samples tested, number of influenza viruses detected and detailed virus characterization results.

**Season**	**2016/2017**	**2017/2018**	**2018/2019**
N samples tested	4,426	6,873	2,047
N viruses detected	1,042	2,334	587
N patients fulfilling study inclusion criteria for VE estimates (N cases/N controls)	767 (442/325)	1,198 (756/442)	1,166 (721/445)
**Typing/genotyping results:**			
**N A(H1N1)pdm09 positive**	21	665	326
N A(H1N1)pdm09 genotyped	14	112	112
A(H1N1)pdm09 genotyping results:			
N 6B	14		
N 6B.1A		99	6
N 6B.1A5		13	66
N 6B.1A6			7
N 6B.1A7			33
**N A(H3N2) positive**	994	137	154
N A(H3N2) genotyped	177	36	90
A(H3N2) genotyping results:			
N 3C.2a	50	26	
N 3C.2a1	104	10	
N 3C.2a1b	12		76 (20^a^)
N 3C.2a2	5		2 (1^b^)
N 3C.2a3	1		2 (1^c^)
N 3C.2a4	5		2 (2^d^)
N 3C.3a	0		8
**N Influenza B positive**	25	1,472	2
N Influenza B genotyped	16	132	2
Influenza B genotyping results:			
N Yamagata genetic clade 3	14	132	2
N Victoria	2		

a *N subclade reassortant HA: 3C.2a1b, NA: 3C.2a2*.

b *N subclade reassortant HA: 3C.2a2, NA: 3C.2a1b*.

c *N subclade reassortant HA: 3C.2a3, NA: 3C.2a2*.

d *N subclade reassortant HA: 3C.2a4, NA: 3C.2a1*.

This table further demonstrates the great diversity and complexity in the pattern of the different circulating influenza virus lineages, and genetic clades and subclades observed during the three seasons. Of special interest was the emergence of HA-NA subclade reassortants of the A(H3N2) viruses during the season 2018/19 (Table 1). Overall, HA-NA subclade reassortants were detected in 24 of the 90 A(H3N2)viruses analyzed by sequencing during this season.

### Vaccine Effectiveness

Generally, in Austria the influenza vaccine coverage is traditionally very low and ranged during the three seasons constantly at a low level between 6 and 7% (2016/17: 7.2%, 2017/18: 6.1%, and 2018/19: 5.9%).

Detailed information on the number of vaccinees and controls as well as the overall and type/subtype specific IVE estimates during the three seasons and the different age groups are provided in Tables 2A–C.

Table 2Number of cases and controls (vaccinated and unvaccinated) and influenza vaccine effectiveness (IVE) adjusted for sex, age, and comorbidity for the seasons 2016/17 (**A**), 2017/18 (**B**, including additionally also IVE estimates for QIV) and 2018/19 (**C**); n.d., not done; QIV, quadrivalent inactivated influenza vaccines; CI, confidence interval.

**Table:** 

**(A)**				
**Season 2016/17**	**Controls**	**Cases**	**adjusted IVE**	
	**Vacc/unvacc**	**Vacc/unvacc**	**IVE (%)**	**CI (95%)**
**Influenza A(H3N2)**, ***N*** **=** **767**			**adj. sex, (age), comorbidity**	
All patients	20/305	37/405	−26	−128 to 31
0–14	1/49	3/89	−65	−1,531 to 83
15–64	12/212	17/242	−7	−131 to 51
65+	7/32	17/55	−25	−264 to 57

**Table:** 

**(B)**								
	**Adjusted IVE estimates (all vaccines)**				**Adjusted IVE estimates (QIV/LAIV only)**			
**Season 2017/18**	**Controls**	**Cases**	**Adjusted IVE**		**Controls**	**Cases**	**Adjusted IVE**	
	**Vacc/unvacc**	**Vacc/unvacc**	**IVE (%)**	**CI(95%)**	**Vacc/unvacc**	**Vacc/unvacc**	**IVE (%)**	**CI (95%)**
**Any influenza**, ***N*** **=** **1,198**			**adj. sex, (age), comorbidity**			**adj. sex, (age), comorbidity**		
All patients	26/416	30/726	39	−5 to 65	10/416	10/726	46	−30 to 78
0–14	9/181	5/287	65	−6 to 88	4/181	3/287	73	−28 to 94
15–64	14/219	21/414	19	−63 to 60	6/219	6/414	47	−69 to 83
65+	3/16	4/25	15	−333 to 83	0/16	1/25	n.d	
**Influenza A(H1N1)pdm09**, ***N*** **=** **705**			**adj. sex, (age), comorbidity**			**adj. sex, (age), comorbidity**		
All patients	26/416	11/252	25	−56 to 64	10/416	2/252	66	−56 to 93
0–14	9/181	1/133	85	−23 to 98	4/181	1/133	72	−163 to 97
15–64	14/219	9/117	−19	−185 to 50	6/219	1/117	68	−172 to 96
65+	3/16	1/2	22	−1,632 to 96	0/16	0/2	n.d.	
**Influenza B**, ***N*** **=** **935**			**adj. sex, (age), comorbidity**			**adj. sex, (age), comorbidity**		
All patients	26/416	19/474	45	−2 to 70	10/416	8/474	40	−56 to 77
0–14	9/181	4/154	63	−38 to 90	4/181	2/154	76	−45 to 96
15–64	14/219	12/297	39	−36 to 72	6/219	5/297	41	−98 to 82
65+	3/16	3/23	29	−395 to 90	0/16	1/23	n.d

**Table:** 

**(C)**				
**Season 2018/19**	**Controls**	**Cases**	**Adjusted IVE**	
	**Vacc/unvacc**	**Vacc/unvacc**	**IVE (%)**	**CI (95%)**
**Any influenza**, ***N*** **=** **1,166**			**adj. sex, (age), comorbidity**	
All patients	66/655	18/427	63	36 to 79
0–14	34/321	4/156	73	20 to 91
15–64	26/316	11/251	51	−2 to 76
65+	6/18	3/20	56	−105 to 90
**A(H1N1)pdm09**, ***N*** **=** **1,017**			**adj. sex, (age), comorbidity**	
All patients	66/655	11/285	65	32 to 82
0–14	34/321	4/111	64	−5 to 88
15–64	26/316	6/164	63	7 to 85
65+	6/18	1/10	69	−196 to 97
**Influenza A(H3N2)**, ***N*** **=** **868**			**adj. sex, (age), comorbidity**	
All patients	66/655	7/140	58	4 to 81
0–14	34/321	0/44	82	−14 to 100
15–64	26/316	5/86	21	−115 to 71
65+	6/18	2/10	43	−244 to 91

**Figure 2:**
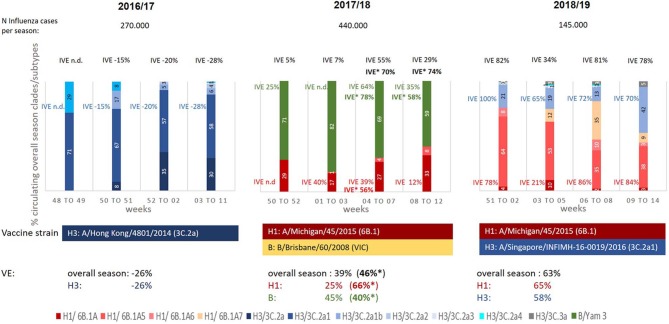
Proportion (%) of circulating influenza virus genetic subclades during seasons 2016/17 to 2018/19; Black: IVE overall, blue: IVE against influenza A(H3N2), red: IVE against influenza A(H1N1)pdm09, green: IVE against Influenza B (aTIV/TIV/QIV and LAIV), bold*: IVEs using quadrivalent vaccines (QIV/LAIV) (Season 2017/18); n.d., not done due to low case numbers.

Overall IVE estimates of season 2016/17, dominated by A(H3N2) viruses of the subclade 3C.2a1 (59% of the viruses analyzed by genotyping), was −26%. This lack of protection of the seasonal vaccine was observed in all age groups (Table 2A) and can be explained by the distinct mismatch between the vaccine strain (genetic clade 3C.2a) and the circulating strains of the different genetic subclades 3C.2a1, 3C.2a4, 3C.2a2, 3C.2a3 (Table 1, Figure 2).

In contrast to the previous one in the following season 2017/18, dominated by influenza B viruses with a co-circulation of A(H1N1)pdm09 strains, an overall IVE of 39% [A(H1N1)pdm09 25%, influenza B 45%] was observed (Table 2B). This far from satisfying overall IVE can be explained by the B-lineage mismatch between TIV/aTIV vaccine strain (Victoria lineage) and the circulating influenza B strains (Yamagata lineage; Figure 2). Despite the low overall IVE and the pronounced influenza B lineage mismatch of the TIV/aTIV vaccine strain a quite good IVE against influenza B viruses of 63% could be observed in children aged 6 month to 14 years. Analyzing IVE for the QIVs, which were available in Austria for the first time during this season, revealed a slight increased overall IVE of 46% and an IVE of 76% for children between 6 month and 14 years of age.

Despite the quite good match between the circulating A(H1N1)pdm09 strains (drift variant clade 6B.1A) and the vaccine strain (clade 6B.1) (Figure 2), the suboptimal IVE of 25% against the A(H1N1)pdm09 can be explained by the low A(H1N1)pdm09 activity in this season (26% of circulating viruses) resulting in a low number of cases.

In contrast, a considerable better IVE of 65% against A(H1N1)pdm09 viruses was obtained in the following season 2018/19 where A(H1N1)pdm09 viruses (again genetic clade 6B.1) were dominating (Table 2). Due to the good match between vaccine strain and circulating strains a very satisfying A(H1N1)pdm specific IVE could be observed for all age groups, especially also for those above the age of 65 years (IVE 69%, Table 2C). During this season an increasing circulation of A(H3N2) viruses could be detected with a relatively good match between the circulating strains (3C.2a1b) and the vaccine strains (3C.2a1, Figure 2), resulting in an overall A(H3N2) specific IVE of 58% and a IVE of 82% in children aged 6 month to 14 years (Table 2C).

The epidemiologic situation during the three seasons shows clearly the diverse and complex pattern of the circulation of different influenza virus lineages and genetic clades and subclades during a season. This influences the IVE during the season and makes reliable IVE estimates even more difficult, as can be seen in Figure 2. This figure provides an overview on the results of the genetic characterization of the circulating influenza viruses during different time periods of epidemic influenza virus circulation (≥50% of samples influenza virus positive) and their influence on IVE estimates.

## Discussion

This paper presents data obtained by the Austrian sentinel surveillance system on the evolution of influenza viruses during the seasons 2016/17 to 2018/19 and the impact of genetic drift on influenza IVE. IVE estimates were carried out using the test-negative case-control design, which currently represents the gold standard study design for IVE studies. The test-negative case-control design is predicated on the assumption that vaccinated persons have the same likelihood of being exposed to influenza as non-vaccinated persons, and that vaccinated and unvaccinated have the same healthcare-seeking behavior, and that sampling of respiratory specimens is performed with equal frequency in both groups (11). Even though, statistical adjustments for different patient groups (age, sex, comorbidities, …) have been performed in our study, an influence of healthcare-seeking behavior on IVE estimates, cannot be completely ruled out, especially with regard to the low vaccination coverage in Austria. Nevertheless, if there would be such a bias, there should be a difference in the clinical presentations of vaccinated and unvaccinated cases. Comparing clinical signs and symptoms in vaccinated and unvaccinated patients in the different seasons revealed no statistical significant difference between these two groups.

The circulation of a heterogeneous mix of influenza virus strains of different types, subtypes and genetic subclades with varying matches to the vaccine strains was typical for each of the three influenza epidemics, substantially reducing IVE.

The **2016/17 influenza** season differed from the other two seasons by the absolute dominance of one specific influenza A subtype and its evolving subclades (Table 1, Figure 2). Such dominance of a single subtype is only very rarely observed. Sequence analyses revealed substantial heterogeneity in the circulating influenza viruses and showed the emergence of the A(H3N2) genetic subclade 3C.2a1 and its drift variants, not only in Europe (12) but worldwide (13), revealing an antigenic mismatch to the vaccine strain. The proportion of this newly evolved subclade of the circulating viruses showed regional differences, depending on the start of the influenza season in the different European regions. In the northern parts of Europe, in Sweden and Finland, the influenza season 2016/17 started early in weeks 47/2016 to 49/2016 and peaked already in week 52/2016 (14). In these countries the genetic influenza surveillance revealed the presence of the genetic A(H3N2) subclade 3C.2a1 in 24% of the samples already at the beginning of the season, with an increase to 75% of the circulating viruses in the final weeks 52/2016 to 02/2017 (14). This circulation pattern is in contrast to that observed in Austria, where the influenza season started several weeks later with its peak in week 02/2017. The time dependent spread of the influenza virus activity throughout Europe may explain the differences in the proportion of circulating A(H3N2) genetic subclades in different regions. In Austria 3C.2a1 subclade accounted already for 71% of the circulating influenza viruses at the beginning of the season. This may also partly explain the absence of influenza vaccine protection in Austria, where negative IVEs throughout the season were observed. This differs to data from other countries, where overall IVE's for this season of around 40% have been described (12–15). Beyond viral genomic variation, birth cohort effects, prior vaccination in addition to the epidemic period may account for regional differences in IVE estimates (16, 17). In this context, the low vaccine coverage in Austria in contrast to that of the other European countries has to be taken into consideration. Nevertheless, detailed analyses of IVEs in Finland and Sweden during the season showed a clear decline in IVEs with increasing proportion of circulating 3C.2a1 viruses [IVE 50% at the start of the season with a drop to 30% at the end (14)], indicating a correlation between viral changes and the observed decline of IVE estimates. This also demonstrates the strong influence of the antigenic match on the IVE and argues, at least in this case, against waning of vaccine induced immunity.

In the following **season 2017/18** a wide range of various patterns in the epidemiologic dominance of influenza virus types and subtypes was observed in different parts of the world. While in the US A(H3N2) viruses dominated, a co-circulation of influenza B/Yamagata and A(H3N2) viruses was observed in Canada (18). In Europe the majority of influenza viruses detected were influenza viruses type B. But even in Europe the pattern of circulating strains differed locally. While in UK an equal co-circulation of influenza B viruses and influenza A(H3N2) viruses was observed, in continental Europe influenza B viruses dominated with locally different proportions of co-circulating A(H1N1)pdm09 and/or A(H3N2) viruses. In Austria influenza B viruses accounted for 67% of the circulating viruses with a co-circulation of A(H1N1)pdm09 viruses (29%) and more or less no epidemic activity of the A(H3N2) viruses (4%).

In Austria the observed type-specific IVE of 45% against influenza B virus infections was comparable with international published data where a quite broad range from 25 to 55% was reported (18–20). Despite the pronounced influenza B lineage mismatch of the TIV/aTIV vaccine strain (vaccine strain B/Victoria), still a quite good IVE against the circulating influenza B/Yamagata viruses was observed. This phenomenon can be explained by influenza B lineage cross-protection, where birth-cohort effects induced by differential prime-boost lineage-exposures may play a role in IVE. Cross lineage protection was observed previously in several studies (17, 21). Nevertheless, as expected, in patients vaccinated with quadrivalent vaccines (QIV/LAIV, perfect vaccine match for the influenza B/Yamagata component) an increased overall IVE of 46% and an IVE of 76% for children between 6 month and 14 years of age could be found for this season in Austria. This is in accordance with data described in Canada (18), and differ to those obtained by the UK (19), which show no effectivity for the TIV against influenza B, but excellent effectivity of the LAIV against influenza B (61%) and A(H1N1)pdm09 (91%).

In Austria A good overall A(H1N1)pdm09 specific IVE of 66% and of 72% in children was also found for quadrivalent vaccines (QIV/LAIV), whereas the analysis including all kind of vaccines used (mostly TIV) revealed an influenza A(H1N1)pdm09 specific IVE of only 25% for this season. A possible explanation of this differences in IVE estimates, may be provided by the low vaccine uptake rate in Austria. The low number of vaccinated patients with confirmed A(H1N1)pdm09 infection disproportionally effects subgroup analyses, as reflected by the wide confidence intervals for this subgroup analyses.

This also affects IVE subgroup analyses during the season (Figure 2), where, based on the low numbers of vaccinees, IVE estimates were not possible for the different types/subtypes for each time period.

Influenza B lineage mismatch did not play any role in the following **season 2018/19**, as this season was dominated by influenza A viruses. In Canada (22) and Hong Kong (23) the 2018/19 influenza season was dominated by influenza A(H1N1)pdm09 viruses. In Europe a complex and over the season constantly changing pattern of the circulation of both influenza A virus subtypes was observed. UK reported the co-circulation of both influenza A virus subtypes, while counties on the European continent reported the dominance or co-circulation of either A(H1N1)pdm09 or A(H3N2) viruses. This significant differences in the geographic spread of the two influenza A subtypes and their genetic subclades, may also explain differences in IVE estimates.

In Austria influenza A(H1N1)pdm09 viruses dominated during the season 2018/19 accounting for 2/3 of all influenza virus infections. Notably, an increase in the proportion of circulating influenza A(H3N2) viruses could be observed over time, with 24% of A(H3N2) viruses at the beginning of the season up to 47% A(H3N2) viruses toward the end of the season (see Figure 2).

The genetic surveillance of the circulating A(H3N2) viruses showed the emergence of various subclades, and additionally also temporal differences in the distribution of the A(H3N2) subclades. During the season the high genetic heterogeneity of the A(H3N2) viruses was expressed by the continued co-circulation and diversification into various genetic A(H3N2) subclades and subclusters. Due to the general genetic variability of the influenza viruses and their ability for reassortment, it is not surprising, that various A(H3N2) hemagglutinin (HA)—neuraminidase (NA) subclade reassortants could be detected by the close genetic monitoring carried out in the national influenza surveillance (Table 1). In Austria, out of 90 viruses genetically characterized 24 (27%) were found to be HA-NA subclade reassortants, and in 3 out of 9 vaccinated patients with confirmed A(H3N2) infection, the HA-NA subclade reassortant could be detected. These reassortments represent an additional factor that further complicates IVE estimates, next to the various circulating subclades and the temporal differences in their distribution. Hence, for the 2018/19 influenza season a broad range for the A(H3N2) specific IVE is reported ranging from −39 to 24% in six European studies (24). In these countries only 59% of the circulating A(H3N2) viruses belonged to the subclade 3C.2a1b, with a good match to the vaccine strain. In Austria 84% of the circulating A(H3N2) viruses belonged to the 3C.2a1b subclade resulting in overall A(H3N2) specific IVE of 58%. An especially high IVE of 82% was observed in children and a quite good IVE of 43% in patients above 65 years.

The A(H1N1)pdm09 viruses circulating during the season 2018/19 have evolved over time from their 2009 ancestor and are becoming genetically more variable, but at a slower pace than the A(H3N2) viruses. In Austria, 88% of the A(H1N1)pdm09 viruses genetically characterized represented the A(H1N1)pdm09 genetic subclades 6B.1A5 or 6B.1A7, and differed slightly from the vaccine strain (sublade 6B.1A). Despite these minor genetic changes, still a quite good overall A(H1N1)pdm09 specific IVE of 65% could be observed. This IVE are comparable with that reported in other European and international studies with A(H1N1)pdm09 IVEs ranging from between 46 and 92% (22–24).

In addition to the influenza virus genetic diversity, the degree of epidemic influenza virus circulation also effects IVE estimates. A higher level of epidemic influenza virus circulation is associated with higher exposure rates and provides therefore higher chances for infection to take place. This may lead to lower IVE estimates in seasons with a high degree of influenza virus activity. In contrast, higher IVE estimates may be observed in seasons with a low degree of influenza virus activity. This may explain the relatively good vaccine protection during the milder season 2018/19, where only minor mismatches with the vaccine strains have been observed.

No significant differences between intraseasonal IVE estimates were observed over the three seasons analyzed. Prior studies (5, 25–27) found evidence for the waning of vaccine protection during a single season with increasing time since vaccination. However, waning of protection is not consistently observed in all seasons or populations. As waning of protection is closely linked with antibody decay, the immunogenicity of the vaccine antigen is a crucial factor. Also the patient's individual immune response is closely linked with the patient's individual prime-boost experience resulting in their own and unique “antibody landscape” (28).

As far as agent factors are concerned, the results of the present study indicate that statements on a protective effect of a vaccine against specific strains are only possible for distinct time periods of an influenza season and with an adjustment for the genetic pattern of the circulating influenza viruses. Although, our data indicate a correlation between IVE and the complex dynamics of circulating strains of a flu season, testing for a statistically significant relationship will require the further analysis of a few more influenza seasons.

IVE estimates are challenging and difficult, as they are influenced by multiple factors like the vaccines used, repeat vs. single season vaccination, the patient's individual prime-boost exposure, and the immunogenicity of the vaccine antigen. Nevertheless, one of the most important factors is still the antigenic match between the vaccine strains and the circulating strains.

The results obtained in our study over the three seasons demonstrate the increasingly complex dynamic of the ever changing genetic pattern of the circulating influenza viruses and their influence on IVE estimates. This genetic and antigenic variability extremely complicates the decisions of the WHO on suitable and optimal influenza vaccine strains and underscores the importance of the development and availability of a universal influenza vaccine.

## Data Availability Statement

The datasets generated for this study can be found in the GISAID Database accession numbers (in the Supplementary Materials).

## Ethics Statement

This study was conducted at the Centre of Virology of the Medical University of Vienna, the WHO NIC for Austria. It is a retrospective analysis of viral and epidemiological data of fully anonymized material collected during the annual influenza surveillance within the frame of Austria's Sentinel Physician Surveillance Network. Written informed consent from the participants' legal guardian/next of kin was not required to participate in this study in accordance with the national legislation and the institutional requirements. The study was performed according to the Declaration of Helsinki and the studies involving human participants were reviewed and approved by Ethics committee of the Medical University of Vienna (EK: 1339/2017).

## Author Contributions

MR-F and TP-K: clinical study design, data analysis, first draft and revision of the manuscript. MK: statistical analyses and critical review of manuscript.

### Conflict of Interest

MK is member of the National Immunization Board. The remaining authors declare that the research was conducted in the absence of any commercial or financial relationships that could be construed as a potential conflict of interest.
