# Supplementary material for: Heterogeneity of Circulating Influenza Viruses and Their Impact on Influenza Virus Vaccine Effectiveness During the Influenza Seasons 2016/17 to 2018/19 in Austria
Source: Front Immunol. 2020 Mar 17;11:434. doi: 10.3389/fimmu.2020.00434 (PMC7092378; doi:10.3389/fimmu.2020.00434)
Supplement: Supplementary file 1 [file Data_Sheet_1.DOCX]

The datasets generated for this study can be found in the GISAID Database accession numbers are: Season 2016/17: EPI998894, EPI998892, EPI998890, EPI998888, EPI998886, EPI998884, EPI998881, EPI998879, EPI998875, EPI998873, EPI998838, EPI902428, EPI866614, EPI866612, EPI998877, EPI998895, EPI998893, EPI998891, EPI998889, EPI998887, EPI998885, EPI998882, EPI998880, EPI998876, EPI998874, EPI998857, EPI902429, EPI866615, EPI866613, EPI998878, EPI1045600, EPI1045270, EPI1045254, EPI1045146, EPI1045144, EPI1045142, EPI1045140, EPI1045138, EPI1044230, EPI1044228, EPI1044226, EPI1044224, EPI1044215, EPI1043484, EPI1043306, EPI1043304, EPI1043302, EPI1043300, EPI1043298, EPI1043296, EPI1043294, EPI1043292, EPI1043290, EPI1043156, EPI1040419, EPI1040417, EPI1040415, EPI1040413, EPI1038453, EPI1038334, EPI1038320, EPI1036305, EPI1036303, EPI1036301, EPI1036299, EPI1036297, EPI1036295, EPI1036293, EPI1036291, EPI1036173, EPI1036017, EPI1035880, EPI1035878, EPI1035874, EPI1035814, EPI1035766, EPI1034955, EPI1034717, EPI1034677, EPI1019899, EPI1019897, EPI1019895, EPI1019893, EPI1019891, EPI1019889, EPI1018234, EPI1018232, EPI1018230, EPI1018228, EPI1018226, EPI1018224, EPI1018212, EPI998934, EPI998932, EPI998930, EPI998928, EPI998926, EPI998924, EPI998922, EPI998799, EPI998797, EPI998795, EPI998793, EPI998791, EPI998789, EPI998630, EPI998530, EPI998507, EPI998505, EPI998503, EPI998501, EPI998499, EPI998497, EPI998495, EPI998493, EPI998486, EPI998482, EPI998478, EPI998459, EPI997147, EPI997145, EPI997143, EPI997141, EPI997130, EPI997066, EPI997064, EPI997062, EPI997051, EPI996988, EPI996985, EPI996905, EPI996843, EPI995822, EPI956189, EPI956179, EPI956177, EPI956169, EPI956164, EPI956153, EPI956149, EPI956145, EPI956139, EPI956114, EPI956112, EPI954550, EPI954548, EPI954546, EPI954544, EPI954542, EPI954540, EPI954530, EPI954514, EPI954512, EPI954510, EPI954500, EPI954490, EPI954488, EPI954486, EPI954485, EPI954482, EPI902430, EPI884316, EPI884313, EPI884309, EPI884307, EPI884304, EPI884301, EPI884298, EPI884296, EPI884294, EPI884291, EPI884289, EPI884286, EPI884284, EPI879342, EPI879288, EPI879286, EPI879284, EPI879282, EPI879280, EPI879278, EPI879081, EPI878746, EPI878352, EPI878350, EPI878347, EPI878345, EPI878343, EPI878341, EPI878340, EPI878338, EPI878336, EPI873798, EPI873796, EPI873794, EPI873792, EPI873790, EPI873789, EPI873788, EPI873786, EPI873702, EPI873701, EPI873699, EPI873698, EPI873697, EPI873696, EPI873695, EPI858861, EPI1045601, EPI1045271, EPI1045255, EPI1045147, EPI1045145, EPI1045143, EPI1045141, EPI1045139, EPI1044231, EPI1044229, EPI1044227, EPI1044225, EPI1044217, EPI1043485, EPI1043307, EPI1043305, EPI1043303, EPI1043301, EPI1043299, EPI1043297, EPI1043295, EPI1043293, EPI1043291, EPI1043157, EPI1040420, EPI1040418, EPI1040416, EPI1040414, EPI1038454, EPI1038343, EPI1038321, EPI1036306, EPI1036304, EPI1036302, EPI1036300, EPI1036298, EPI1036296, EPI1036294, EPI1036292, EPI1036174, EPI1036026, EPI1035881, EPI1035879, EPI1035875, EPI1035822, EPI1035767, EPI1034956, EPI1034910, EPI1034678, EPI1019900, EPI1019898, EPI1019896, EPI1019894, EPI1019892, EPI1019890, EPI1018235, EPI1018233, EPI1018231, EPI1018229, EPI1018227, EPI1018225, EPI1018213, EPI998935, EPI998933, EPI998931, EPI998929, EPI998927, EPI998925, EPI998923, EPI998800, EPI998798, EPI998796, EPI998794, EPI998792, EPI998790, EPI998637, EPI998543, EPI998508, EPI998506, EPI998504, EPI998502, EPI998500, EPI998498, EPI998496, EPI998494, EPI998487, EPI998483, EPI998479, EPI998472, EPI997148, EPI997146, EPI997144, EPI997142, EPI997139, EPI997067, EPI997065, EPI997063, EPI997058, EPI996989, EPI996987, EPI996911, EPI996844, EPI995823, EPI956190, EPI956180, EPI956178, EPI956172, EPI956166, EPI956155, EPI956150, EPI956146, EPI956140, EPI956115, EPI956113, EPI954551, EPI954549, EPI954547, EPI954545, EPI954543, EPI954541, EPI954531, EPI954515, EPI954513, EPI954511, EPI954501, EPI954491, EPI954489, EPI954487, EPI954484, EPI954483, EPI902462, EPI884317, EPI884314, EPI884310, EPI884308, EPI884305, EPI884302, EPI884300, EPI884297, EPI884295, EPI884292, EPI884290, EPI884287, EPI884285, EPI879343, EPI881903, EPI879287, EPI879285, EPI879283, EPI879281, EPI879279, EPI879102, EPI878767, EPI878353, EPI878351, EPI878348, EPI878346, EPI878344, EPI878339, EPI878337, EPI873799, EPI873797, EPI873795, EPI873793, EPI873791, EPI873787, EPI873703, EPI998936, EPI873700, EPI887362, EPI998937, EPI998938, EPI998939, EPI858862, EPI1045604, EPI1045602, EPI998920, EPI998918, EPI998915, EPI998913, EPI998911, EPI998909, EPI998906, EPI998904, EPI998902, EPI998900, EPI998898, EPI995820, EPI881234, EPI881232, EPI1045605, EPI1045603, EPI998921, EPI998917, EPI998916, PI998914, EPI998912, EPI998910, EPI998907, EPI998905, EPI998903, EPI998901, EPI998899, EPI995821, EPI881235, EPI881233, Season 2017/18: EPI1200602, EPI1200604, EPI1200606, EPI1200608, EPI1202733, EPI1202735, EPI1202737, EPI1202739, EPI1202741, EPI1202743, EPI1202767, EPI1202769, EPI1202745, EPI1202795, EPI1202771, EPI1202747, EPI1202749, EPI1202773, EPI1202751, EPI1202753, EPI1202775, EPI1202777, EPI1202755, EPI1202779, EPI1202781, EPI1202783, EPI1202757, EPI1202785, EPI1202759, EPI1202787, EPI1202789, EPI1202761, EPI1202763, EPI1202791, EPI1202765, EPI1202793, EPI1251603, EPI1251605, EPI1203476, EPI1203472, EPI1203423, EPI1251607, EPI1203474, EPI1203478, EPI1251609, EPI1214125, EPI1214157, EPI1251613, EPI1214135, EPI1251619, EPI1251621, EPI1214143, EPI1214137, EPI1251635, EPI1251631, EPI1251633, EPI1214149, EPI1251611, EPI1251617, EPI1251623, EPI1243521, EPI1251643, EPI1251601, EPI1243515, EPI1251647, EPI1244156, EPI1251649, EPI1251651, EPI1243529, EPI1244164, EPI1251653, EPI1251625, EPI1251655, EPI1243535, EPI1251629, EPI1251637, EPI1244142, EPI1251641, EPI1251627, EPI1251615, EPI1243519, EPI1251639, EPI1243523, EPI1251645, EPI1244172, EPI1251657, EPI1244174, EPI1274631, EPI1274635, EPI1274645, EPI1274651, EPI1274665, EPI1274673, EPI1274677, EPI1274681, EPI1274683, EPI1274687, EPI1274691, EPI1274693, EPI1274699, EPI1274701, EPI1274705, EPI1274707, EPI1200601, EPI1200603, EPI1200605, EPI1200607, EPI1202732, EPI1202734, EPI1202736, EPI1202738, EPI1202740, EPI1202742, EPI1202766, EPI1202768, EPI1202744, EPI1202794, EPI1202770, EPI1202746, EPI1202748, EPI1202772, EPI1202750, EPI1202752, EPI1202774, EPI1202776, EPI1202754, EPI1202778, EPI1202780, EPI1202782, EPI1202756, EPI1202784, EPI1202758, EPI1202786, EPI1202788, EPI1202760, EPI1202762, EPI1202790, EPI1202764, EPI1202792, EPI1251602, EPI1251604, EPI1203475, EPI1203471, EPI1203422, EPI1251606, EPI1203473, EPI1203477, EPI1251608, EPI1214124, EPI1214156, EPI1251612, EPI1214134, EPI1251618, EPI1251620, EPI1214142, EPI1214136, EPI1251634, EPI1251630, EPI1251632, EPI1214148, EPI1251610, EPI1251616, EPI1251622, EPI1243520, EPI1251642, EPI1251658, EPI1243514, EPI1251646, EPI1244155, EPI1251648, EPI1251650, EPI1243528, EPI1244163, EPI1251652, EPI1251624, EPI1251654, EPI1243534, EPI1251628, EPI1251636, EPI1244141, EPI1251640, EPI1251626, EPI1251614, EPI1243518, EPI1251638, EPI1243522, EPI1251644, EPI1244171, EPI1251656, EPI1244173, EPI1274630, EPI1274634, EPI1274644, EPI1274650, EPI1274664, EPI1274672, EPI1274676, EPI1274680, EPI1274682, EPI1274686, EPI1274690, EPI1274692, EPI1274698, EPI1274700, EPI1274704, EPI1274706, EPI1203470, EPI1214117, EPI1203421, EPI1255963, EPI1203466, EPI1255957, EPI1255979, EPI1255955, EPI1203462, EPI1255985, EPI1203460, EPI1255983, EPI1255965, EPI1255961, EPI1203468, EPI1255987, EPI1203464, EPI1255981, EPI1214119, EPI1255959, EPI1255969, EPI1255971, EPI1255967, EPI1255975, EPI1244126, EPI1214147, EPI1214151, EPI1244138, EPI1255973, EPI1244116, EPI1255977, EPI1244168, EPI1243517, EPI1203469, EPI1214116, EPI1203420, EPI1255962, EPI1203465, EPI1255956, EPI1255978, EPI1255954, EPI1203461, EPI1255984, EPI1203459, EPI1255982, EPI1255964, EPI1255960, EPI1203467, EPI1255986, EPI1203463, EPI1255980, EPI1214118, EPI1255958, EPI1255968, EPI1255970, EPI1255966, EPI1255974, EPI1244125, EPI1214146, EPI1214150, EPI1244137, EPI1255972, EPI1244115, EPI1255976, EPI1244167, EPI1243516, EPI1259901, EPI1259905, EPI1214115, EPI1202991, EPI1259885, EPI1202985, EPI1214109, EPI1259887, EPI1259857, EPI1202965, EPI1214107, EPI1214113, EPI1259881, EPI1202969, EPI1214111, EPI1259877, EPI1259863, EPI1202989, EPI1259871, EPI1259855, EPI1202967, EPI1202975, EPI1259909, EPI1259903, EPI1259847, EPI1259849, EPI1259861, EPI1259867, EPI1259891, EPI1202963, EPI1203418, EPI1202981, EPI1214099, EPI1202971, EPI1203419, EPI1259865, EPI1202973, EPI1259897, EPI1259845, EPI1214101, EPI1202977, EPI1259875, EPI1202983, EPI1259851, EPI1214105, EPI1259869, EPI1259895, EPI1202993, EPI1259853, EPI1259883, EPI1259893, EPI1259873, EPI1259907, EPI1259859, EPI1259889, EPI1202979, EPI1259921, EPI1259919, EPI1244110, EPI1259879, EPI1214103, EPI1259917, EPI1259913, EPI1259911, EPI1259915, EPI1202987, EPI1202995, EPI1214155, EPI1214121, EPI1259925, EPI1214127, EPI1214129, EPI1214133, EPI1244108, EPI1214131, EPI1214139, EPI1214141, EPI1244122, EPI1214153, EPI1259931, EPI1244120, EPI1244170, EPI1244128, EPI1214123, EPI1259927, EPI1244124, EPI1259933, EPI1214145, EPI1259935, EPI1244134, EPI1244136, EPI1259937, EPI1244144, EPI1259947, EPI1244132, EPI1259941, EPI1244140, EPI1259943, EPI1244152, EPI1259929, EPI1244154, EPI1244112, EPI1244114, EPI1259923, EPI1243525, EPI1243527, EPI1244162, EPI1259949, EPI1259953, EPI1244166, EPI1244158, EPI1244160, EPI1259951, EPI1243533, EPI1244150, EPI1243531, EPI1243537, EPI1244118, EPI1243513, EPI1244130, EPI1259939, EPI1259945, EPI1244146, EPI1244148, EPI1259899, EPI1243539, EPI1243541, EPI1274617, EPI1274619, EPI1274621, EPI1274623, EPI1274625, EPI1274627, EPI1274629, EPI1274633, EPI1274637, EPI1274639, EPI1274641, EPI1274643, EPI1274647, EPI1274649, EPI1274653, EPI1274655, EPI1274657, EPI1274659, EPI1274661, EPI1274663, EPI1274667, EPI1274669, EPI1274671, EPI1274675, EPI1274679, EPI1274685, EPI1274689, EPI1274695, EPI1274697, EPI1274703, EPI1259900, EPI1259904, EPI1214114, EPI1202990, EPI1259884, EPI1202984, EPI1214108, EPI1259886, EPI1259856, EPI1202964, EPI1214106, EPI1214112, EPI1259880, EPI1202968, EPI1214110, EPI1259876, EPI1259862, EPI1202988, EPI1259870, EPI1259854, EPI1202966, EPI1202974, EPI1259908, EPI1259902, EPI1259846, EPI1259848, EPI1259860, EPI1259866, EPI1259890, EPI1202962, EPI1202980, EPI1214098, EPI1202970, EPI1259864, EPI1202972, EPI1259896, EPI1259844, EPI1214100, EPI1202976, EPI1259874, EPI1202982, EPI1259850, EPI1214104, EPI1259868, EPI1259894, EPI1202992, EPI1259852, EPI1259882, EPI1259892, EPI1259872, EPI1259906, EPI1259858, EPI1259888, EPI1202978, EPI1259920, EPI1259918, EPI1244109, EPI1259878, EPI1214102, EPI1259916, EPI1259912, EPI1259910, EPI1259914, EPI1202986, EPI1202994, EPI1214154, EPI1214120, EPI1259924, EPI1214126, EPI1214128, EPI1214132, EPI1244107, EPI1214130, EPI1214138, EPI1214140, EPI1244121, EPI1214152, EPI1259930, EPI1244119, EPI1244169, EPI1244127, EPI1214122, EPI1259926, EPI1244123, EPI1259932, EPI1214144, EPI1259934, EPI1244133, EPI1244135, EPI1259936, EPI1244143, EPI1259946, EPI1244131, EPI1259940, EPI1244139, EPI1259942, EPI1244151, EPI1259928, EPI1244153, EPI1244111, EPI1244113, EPI1259922, EPI1243524, EPI1243526, EPI1244161, EPI1259948, EPI1259952, EPI1244165, EPI1244157, EPI1244159, EPI1259950, EPI1243532, EPI1244149, EPI1243530, EPI1243536, EPI1244117, EPI1243512, EPI1244129, EPI1259938, EPI1259944, EPI1244145, EPI1244147, EPI1259898, EPI1243538, EPI1243540, EPI1274616, EPI1274618, EPI1274620, EPI1274622, EPI1274624, EPI1274626, EPI1274628, EPI1274632, EPI1274636, EPI1274638, EPI1274640, EPI1274642, EPI1274646, EPI1274648, EPI1274652, EPI1274654, EPI1274656, EPI1274658, EPI1274660, EPI1274662, EPI1274666, EPI1274668, EPI1274670, EPI1274674, EPI1274678, EPI1274684, EPI1274688, EPI1274694, EPI1274696, EPI1274702, Season 2018/19: EPI1324903, EPI1327457, EPI1351449, EPI1351455, EPI1351463, EPI1351465, EPI1351475, EPI1351477, EPI1351483, EPI1351487, EPI1351491, EPI1351493, EPI1351489, EPI1364883, EPI1364887, EPI1364893, EPI1364895, EPI1364907, EPI1364915, EPI1364917, EPI1364889, EPI1364911, EPI1364899, EPI1364921, EPI1381442, EPI1381444, EPI1381448, EPI1381460, EPI1381456, EPI1381454, EPI1381450, EPI1381452, EPI1381446, EPI1381464, EPI1381458, EPI1381476, EPI1381440, EPI1381468, EPI1381482, EPI1381486, EPI1381478, EPI1381516, EPI1381480, EPI1381484, EPI1381500, EPI1381494, EPI1381518, EPI1381506, EPI1381492, EPI1381498, EPI1381512, EPI1381514, EPI1381519, EPI1381525, EPI1381527, EPI1381496, EPI1381512, EPI1398458, EPI1398460, EPI1398462, EPI1398468, EPI1398454, EPI1398464, EPI1398446, EPI1398448, EPI1398456, EPI1398434, EPI1398436, EPI1398450, EPI1398452, EPI1398470, EPI1398472, EPI1398476, EPI1398482, EPI1398484, EPI1398488, EPI1398492, EPI1398474, EPI1398478, EPI1398486, EPI1398440, EPI1398442, EPI1398496, EPI1398498, EPI1398500, EPI1420386, EPI1420401, EPI1420403, EPI1420409, EPI1420418, EPI1420426, EPI1420388, EPI1420390, EPI1420392, EPI1420396, EPI1420399, EPI1420432, EPI1420441, EPI1420443, EPI1440524, EPI1440558, EPI1440560, EPI1440566, EPI1440530, EPI1440548, EPI1440546, EPI1505613, EPI1505615, EPI1505617, EPI1505619, EPI1595473, EPI1595475, EPI1327458, EPI1351448, EPI1351454, EPI1351462, EPI1351464, EPI1351474, EPI1351476, EPI1351482, EPI1351486, EPI1351490, EPI1351492, EPI1351488, EPI1364882, EPI1364886, EPI1364892, EPI1364894, EPI1364906, EPI1364914, EPI1364916, EPI1364888, EPI1364910, EPI1364898, EPI1364920, EPI1381441, EPI1381443, EPI1381447, EPI1381459, EPI1381455, EPI1381453, EPI1381449, EPI1381451, EPI1381445, EPI1381463, EPI1381457, EPI1381475, EPI1381439, EPI1381467, EPI1381481, EPI1381485, EPI1381477, EPI1381515, EPI1381479, EPI1381483, EPI1381499, EPI1381493, EPI1381517, EPI1381505, EPI1381491, EPI1381497, EPI1381511, EPI1381513, EPI1381524, EPI1381526, EPI1381495, EPI1398457, EPI1398459, EPI1398461, EPI1398467, EPI1398453, EPI1398463, EPI1398445, EPI1398447, EPI1398455, EPI1398433, EPI1398435, EPI1398449, EPI1398451, EPI1398469, EPI1398471, EPI1398475, EPI1398481, EPI1398483, EPI1398487, EPI1398491, EPI1398473, EPI1398477, EPI1398485, EPI1398439, EPI1398441, EPI1398495, EPI1398497, EPI1398499, EPI1420385, EPI1420400, EPI1420402, EPI1420408, EPI1420417, EPI1420425, EPI1420387, EPI1420389, EPI1420391, EPI1420395, EPI1420398, EPI1420431, EPI1420440, EPI1420442, EPI1440523, EPI1440557, EPI1440559, EPI1440565, EPI1440529, EPI1440547, EPI1440545, EPI1505614, EPI1505616, EPI1505618, EPI1505620, EPI1595474, EPI1595476, EPI1321482, EPI1324901, EPI1327455, EPI1328834, EPI1336511, EPI1336513, EPI1351451, EPI1351453, EPI1351457, EPI1351459, EPI1351461, EPI1351467, EPI1351471, EPI1351473, EPI1351479, EPI1351469, EPI1351481, EPI1351485, EPI1351495, EPI1364901, EPI1364909, EPI1364913, EPI1364885, EPI1364891, EPI1364903, EPI1364905, EPI1364897, EPI1364923, EPI1364919, EPI1364925, EPI1364927, EPI1381466, EPI1381462, EPI1381472, EPI1381470, EPI1381474, EPI1381488, EPI1381508, EPI1381502, EPI1381504, EPI1381490, EPI1381523, EPI1381521, EPI1381510, EPI1398466, EPI1398480, EPI1398490, EPI1398438, EPI1398444, EPI1398494, EPI1398502, EPI1398504, EPI1420405, EPI1420407, EPI1420411, EPI1420413, EPI1420415, EPI1420420, EPI1420422, EPI1420394, EPI1420424, EPI1420430, EPI1420435, EPI1420437, EPI1420439, EPI1420445, EPI1420447, EPI1420449, EPI1440562, EPI1440564, EPI1440526, EPI1440532, EPI1440538, EPI1440534, EPI1440536, EPI1440552, EPI1440542, EPI1440544, EPI1440554, EPI1440556, EPI1440528, EPI1440540, EPI1440550, EPI1505621, EPI1505623, EPI1505625, EPI1321483, EPI1324902, EPI1327456, EPI1328835, EPI1336512, EPI1336514, EPI1351450, EPI1351452, EPI1351456, EPI1351458, EPI1351460, EPI1351466, EPI1351470, EPI1351472, EPI1351478, EPI1351468, EPI1351480, EPI1351484, EPI1351494, EPI1364900, EPI1364908, EPI1364912, EPI1364884, EPI1364890, EPI1364902, EPI1364904, EPI1364896, EPI1364922, EPI1364918, EPI1364924, EPI1364926, EPI1381465, EPI1381461, EPI1381471, EPI1381469, EPI1381473, EPI1381487, EPI1381507, EPI1381501, EPI1381503, EPI1381489, EPI1381522, EPI1381520, EPI1381509, EPI1398465, EPI1398479, EPI1398489, EPI1398437, EPI1398443, EPI1398493, EPI1398501, EPI1398503, EPI1420404, EPI1420406, EPI1420410, EPI1420412, EPI1420414, EPI1420419, EPI1420421, EPI1420393, EPI1420423, EPI1420429, EPI1420433, EPI1420436, EPI1420438, EPI1420444, EPI1420446, EPI1420448, EPI1440561, EPI1440563, EPI1440525, EPI1440531, EPI1440537, EPI1440533, EPI1440535, EPI1440551, EPI1440541, EPI1440543, EPI1440553, EPI1440555, EPI1440527, EPI1440539, EPI1440549, EPI1505622, EPI1505624, EPI1505626, EPI1351497, EPI1420428, EPI1351496, EPI1420427
